# Inflammatory biomarkers after an exercise intervention in childhood acute lymphoblastic leukemia survivors

**DOI:** 10.1002/jha2.588

**Published:** 2022-09-29

**Authors:** Tuomas Lähteenmäki Taalas, Liisa Järvelä, Harri Niinikoski, Anu Huurre, Arja Harila‐Saari

**Affiliations:** ^1^ University of Turku Turku Finland; ^2^ Department of Women's and Children's Health Uppsala University Uppsala Sweden; ^3^ Department of Pediatrics and Adolescent Medicine Turku University Hospital Turku Finland

**Keywords:** ALL, endothelial dysfunction, exercise intervention, inflammation, insulin resistance, proteomic

## Abstract

Cancer survivors show increased risk for non‐communicable diseases and chronic low‐grade inflammation characterizes the development of such diseases. We investigated inflammatory plasma protein profiles of survivors of childhood acute lymphoblastic leukemia (ALL) in comparison to healthy controls and after an intervention with a home‐based exercise program. Survivors of childhood ALL aged 16–30 years (*n* = 21) with a median age at diagnosis 4.9 (1.6–12.9) years and a median time of 15.9 years from diagnosis, and sex‐ and age‐matched healthy controls (*n* = 21) were studied. Stored plasma samples were analyzed with Olink's 92‐protein‐wide Inflammation panel in 21 ALL long‐term survivors at baseline, after a previous 16‐week home‐based exercise intervention (*n* = 17) and in 21 age‐ and sex‐matched controls at baseline. Protein expression levels were compared between the groups. Inflammatory protein levels did not differ between the survivors and controls at baseline. Significantly reduced levels after the intervention were found in 11 proteins related to either vascular inflammation, insulin resistance, or both: tumor necrosis factor superfamily member 14 (TNFSF14), oncostatin M (OSM), monocyte chemoattractant protein 1 (MCP‐1), MCP‐2, fibroblast growth factor 21 (FGF‐21), chemokine (C‐C motif) ligand 4 (CCL4), transforming growth factor alpha (TGF‐α), tumor necrosis factor‐related apoptosis‐inducing ligand 10 (TRAIL), adenosine deaminase (ADA), chemokine (C‐X‐C motif) ligand 6 (CXCL6), and latency‐associated peptide transforming growth factor beta 1 (LAP TGF‐β1). The ALL survivors were not significantly more affected by inflammation than controls at baseline. The survivors’ 16‐week exercise intervention led to significant reduction in inflammatory protein levels. Physical exercise should be promoted for survivors of childhood cancer.

**Abbreviations**
ADA: adenosine deaminase
ALL: acute lymphoblastic leukemia
BMI: body mass index
CCL: chemokine (C‐C motif) ligand
CCR: C‐C chemokine receptor
CI: chronotropic insufficiency
CVD: cardiovascular disease
CXCL: chemokine (C‐X‐C motif) ligand
FGF‐21: fibroblast growth factor 21
IR: insulin resistance
LAP TGF‐β1: latency‐associated peptide transforming growth factor beta 1
MCP: monocyte chemoattractant protein
MetS: metabolic syndrome
NOPHO: Nordic Society for Paediatric Haematology and Oncology
NPX: Normalized Protein eXpression
OSM: oncostatin M
TGF‐α: transforming growth factor alpha
TGF‐β: transforming growth factor beta
TNFSF14/LIGHT: tumor necrosis factor superfamily member 14
TRAIL/TNFSF10: tumor necrosis factor‐related apoptosis‐inducing ligand 10
VO_2_
: oxygen uptake

## 1 INTRODUCTION

Acute lymphoblastic leukemia (ALL) is the most common malignancy in childhood with a 5‐year survival rate of over 90% in many high‐income countries [1, 2]. Unfortunately, due to ever more effective and intensive treatment protocols, the growing population of survivors tend to suffer from serious acute and late adverse effects [3, 4, 5, 6]. ALL survivors are at an increased risk for premature development of non‐communicable diseases such as emerging metabolic syndrome (MetS) and cardiovascular disease (CVD) [7]. Survivors of childhood ALL have recently been shown to be able to complete a maximal cardiopulmonary exercise test (i.e., achieve peak oxygen uptake [VO_2_], maximal oxygen consumption) without being limited by their symptoms [8]. However, most of them (65.7%) did not achieve their age‐predicted heart rate maximum, and some (6.9%) presented chronotropic incompetence (CI). In addition, 35.8% of the 216 ALL survivors studied were at high risk of developing CI [9]. CI is common in patients with CVD, as it produces exercise intolerance and is an independent predictor of adverse cardiovascular events and mortality [10]. In the St. Jude Lifetime Study Cohort, there was evidence of exercise intolerance (VO_2peak_ <85% of predicted) in majority of the childhood cancer survivors [11]. Exercise intolerance was significantly more common among survivors than healthy controls, and CI was associated with exercise intolerance whereas, for example, an abnormal ejection fraction (<53%) was not [11]. Our previous study also presented worse VO_2peak_ in survivors of ALL than in healthy controls [12], and especially the female survivors had inferior fitness and physical activity levels, which is reported and discussed by others as well [13, 14]. Especially, the female survivors of ALL have long been known to have a higher risk of late cardiotoxic and other treatment‐related toxic effects than males [15, 16]. Because of previously partly scarce data on the late effects of common Nordic ALL‐treatment protocols used since 1986, we have studied in 2007–2008 the fitness [12] and metabolic risk factors [17], as well as, endothelial [18] and cardiac function [19], in a cohort of childhood ALL survivors and healthy controls. The effect of a home‐based exercise intervention on these factors were studied as well among the survivors.

Chronic low‐grade inflammation, a hallmark of aging, is widely considered to be an underlying factor in the development of non‐communicable diseases [20, 21, 22, 23]. Studies on epigenetics, telomere length, and chronic inflammation have shown that ALL survivors are in fact aging in an accelerated way [24, 25, 26, 27]. Chronic inflammation can be reduced by regular moderate exercise and the beneficial health effects may be due to favorable epigenetic changes, as discussed in a review article [28]. Protein expression studies, or expression proteomics, have revealed that the changes that happen with aging in one's proteome correlate with the protein profiles in age‐related diseases, such as CVD [29]. Besides aging, elevated levels of inflammatory proteins are also associated with excess adipose tissue and insulin resistance (IR) [30, 31], which are considered catalysts in developing the full spectrum of MetS traits [32]. To mitigate the inflammatory burden, even a short 2‐week high‐intensity intermittent training program has been shown to be effective in an overweight and obese male cohort [30]. Our home‐based 16‐week exercise intervention among long‐term childhood ALL survivors improved their cardiovascular health in general and more specifically improved IR and fitness (VO_2peak_), decreased waist circumference [17], and improved attenuated left ventricle diastolic function [19] as well as endothelial structure and function [18]. One review article highlighted the broad beneficial anti‐inflammatory effects of regular exercise training even after adjustment for potential confounders such as body mass index (BMI), and indicated that habitual exercise may be capable of delaying immune dysregulation and immunosenescence, which is associated with aging [33]. In contrast, harmful effects linked to extreme exercise training workloads exist as well [28, 33], but childhood cancer survivors are not likely to partake in extreme exercise as the majority of them report low levels of physical activity [34, 35].

Few studies have used extensive proteomic panels of inflammation markers to explore the process of low‐grade inflammation, and even fewer have done so among ALL survivors. We have seen signs of worse pulmonary and cardiovascular function in long‐term childhood ALL survivors compared to healthy controls, but no effect on humoral metabolic risk factors. We had plasma samples stored from the original study, and we hypothesized that survivors of ALL have unbeneficial inflammatory proteomes and that we can see an improvement in their inflammatory proteome after exercise, which corresponds with the clinical improvements already observed in our previous exercise intervention [17]^–^[19]. Hence, the aim of this study was to fill this knowledge gap regarding the humoral health and the inflammatory proteome by investigating changes after ALL treatment compared to healthy controls and explore the effects of the previous 16‐week home‐based exercise intervention on the inflammatory proteome of survivors of childhood ALL.

## 2 METHODS

**FIGURE 1:**
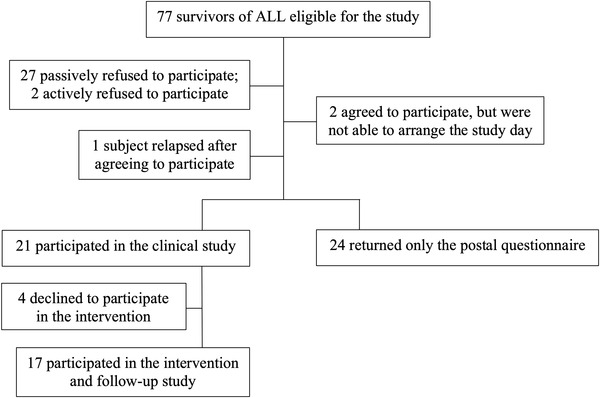
Flow chart of the acute lymphoblastic leukemia (ALL) survivors’ inclusion in the original study [12]. Eligible ALL survivors were identified from local files of the Nordic Society for Paediatric Haematology and Oncology (NOPHO) database with the inclusion criteria of age 16–30 years, age at diagnosis ≤16 years, treatment in Turku or Tampere University Hospital District, diagnosis made 1986 or later, treatment according to the Nordic regimen [66], and first continuous remission without bone‐marrow transplantation. Patients with Down's syndrome were excluded. A postal questionnaire was used to collect information from the non‐participating cohort on, for example, how the participants represent the whole cohort.

This was an add‐on study to a previous non‐randomized controlled intervention cohort study [17, 18], which had a cross‐sectional first phase with controls, and a second phase consisting of an exercise intervention for ALL survivors. Controls did not partake in the intervention because the original study focused on ways to improve the assumed poor cardiovascular health of the survivors in light of such past reports, and because it was practically challenging to accomplish. Stored plasma samples from the original study in Turku, Finland, which compared the cardiac and endothelial health in a cohort of ALL survivors and controls in 2007–2008, were used in this study. The inclusion and exclusion criteria and a flow chart of the survivors’ inclusion in the original study are presented in Figure 1.

### 2.1 Study population and the exercise intervention

**FIGURE 2:**
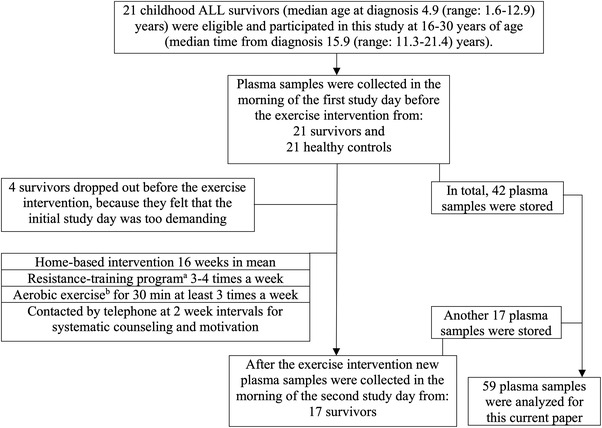
Flow chart of the study population and the exercise intervention as well as timing of the plasma sample collections and clinical examinations on the first and second study days. ^a^An illustrated home muscle‐training program that included eight exercises to strengthen gluteal and lower limb muscles, shoulders and upper limb muscles, abdominal muscles, and back muscles. as many repeats as possible for each of the exercises, and repeat the cycle three times per session. All the subjects were informed and motivated similarly by one person. ^b^The subjects were encouraged to undertake aerobic exercise of their own choice (e.g., walking, jogging, aerobics) at least three times per week for 30 min per session either as a warm‐up for the muscle‐training program or on separate days.

The study population and the intervention have been previously described [12, 17]. In brief, the population consisted of 21 (11 females) long‐term survivors of childhood ALL aged 16–30 years at the time of the original study in 2007–2008. They were diagnosed with ALL between 1986 and 1996 and treated according to the Nordic Society for Paediatric Haematology and Oncology (NOPHO) ALL86 or a later NOPHO protocol. Median age at diagnosis was 4.9 (range: 1.6–12.9) years and median time from diagnosis 15.9 (range: 11.3–21.4) years. Healthy control subjects consisted of 21 (11 females) age‐ and sex‐matched siblings (5/21), friends (11/21), or other non‐athletic adolescents and adults (5/21). Four of the survivors and five of the controls were active smokers and one of the controls used actively snuff tobacco. Two survivors and three controls had a previous history of regular smoking. One survivor smoked occasionally and three controls had smoked occasionally. All the regular or previous regular smokers among the survivors and the controls had smoked for 31.5 and 32.5 years in total, respectively. Alcohol drinking habits were similar in both groups. Thirteen and 15 survivors and controls, respectively, drank alcohol at least once a month, while five survivors and three controls drank alcohol more seldom than once a month. Three persons in both groups did not drink alcohol at all. At baseline, 21 ALL survivors and 21 age‐ and sex‐matched controls underwent a physical examination and provided blood samples. Seventeen of the 21 survivors completed a home‐based exercise intervention [18] of 16 weeks in mean (4/21 survivors dropped out before starting the intervention because they felt that the test day was too demanding). The same parameters as at baseline were collected for these 17 ALL survivors after completion of the exercise intervention. Timing of the sampling and the survivors’ and controls’ examination test days are presented in Figure 2.

The exercise program in 2007–2008 was developed by experts in sports and exercise medicine and exercise science. In brief, the subjects were provided illustrated instructions for the home‐based muscle‐training program and told to perform it three to four times per week. This program consisted of eight exercises to strengthen gluteal and lower limb muscles, shoulders and upper limb muscles, abdominal muscles, and back muscles. The subjects were instructed to do as many repeats as possible for each exercise and repeat the cycle three times per session. They were also encouraged to do 30‐min aerobic exercise sessions of their own choice (e.g., walking, jogging, aerobics) at least three times per week either as a warm‐up or separate to the muscle training. All subjects also received pedometers and information on daily step goals to motivate them to increase physical activity by monitoring the steps taken. Further details have been described earlier [17]. Description of the intervention and the timing of the different stages of the study are presented in Figure 2.

### 2.2 Proximity extension assay

In total, 59 plasma samples stored in Turku and collected in 2007–2008 (*n* = 21 ALL survivors at baseline, *n* = 17 ALL survivors after the intervention, and *n* = 21 controls at baseline) were analyzed for 92 proteins related to inflammation using the Olink Target 96 Inflammation panel (article number 95302, Olink Proteomics, Uppsala, Sweden) at the Clinical Biomarkers Facility at SciLifeLab (Uppsala, Sweden). The samples were thawed, centrifuged, measured, and refrozen to ‐80°C for shipment from Turku to Uppsala according to the company's instructions. The samples were transferred to a 96‐well polymerase chain reaction plate such that they were both randomized and de‐identified. All 59 samples passed quality control.

The concentrations of the proteins analyzed with the Inflammation panel are reported using a unit of relative quantification, which means that the values cannot be compared between different proteins, only between different samples. Because the reported concentrations are relative, they cannot be compared to any clinical reference values, only change of the concentration is relevant. This unit, called Normalized Protein eXpression (NPX), is an arbitrary unit defined by Olink to achieve minimal intra‐ and inter‐assay variation. NPX is on a log2 scale [36].

### 2.3 Statistical analysis

Inflammation panel data in NPX values were analyzed using two paired *t*‐tests, Benjamini–Hochberg method for *p*‐value correction with 5% false discovery rate, and a distribution boxplot (Figure S1) for outlier analysis. *p*‐Values <0.05 were considered statistically significant after correction with the Benjamini–Hochberg method. A principal component analysis plot (Figure S2) assessed possible outlier samples. No outliers were found, and all 59 samples could be included in the paired *t*‐tests, which were performed to compare results of the ALL survivors at baseline versus after intervention, and ALL survivors at baseline versus controls. Only the 17 survivors who completed the assessment both before and after the intervention were included in the paired *t*‐test between them. These analyses were performed using R version 3.6.1. for Windows [37]. For interpretation of results, clinical data from the original study were utilized. The main outcomes of the already earlier published, and now utilized, data are presented in Table S1. In addition, Fischer's exact test (SAS JMP Pro 16.0.0 for Windows) was used to compare the number of survivors and controls with regard to smoking history (not smoking, smoking or using snuff tobacco regularly, history of previous regular smoking, and irregular smoking) and alcohol consumption habits (alcohol use at least once a month, alcohol use less frequently than once a month, and not using alcohol at all).

## 3 RESULTS

**FIGURE 3:**
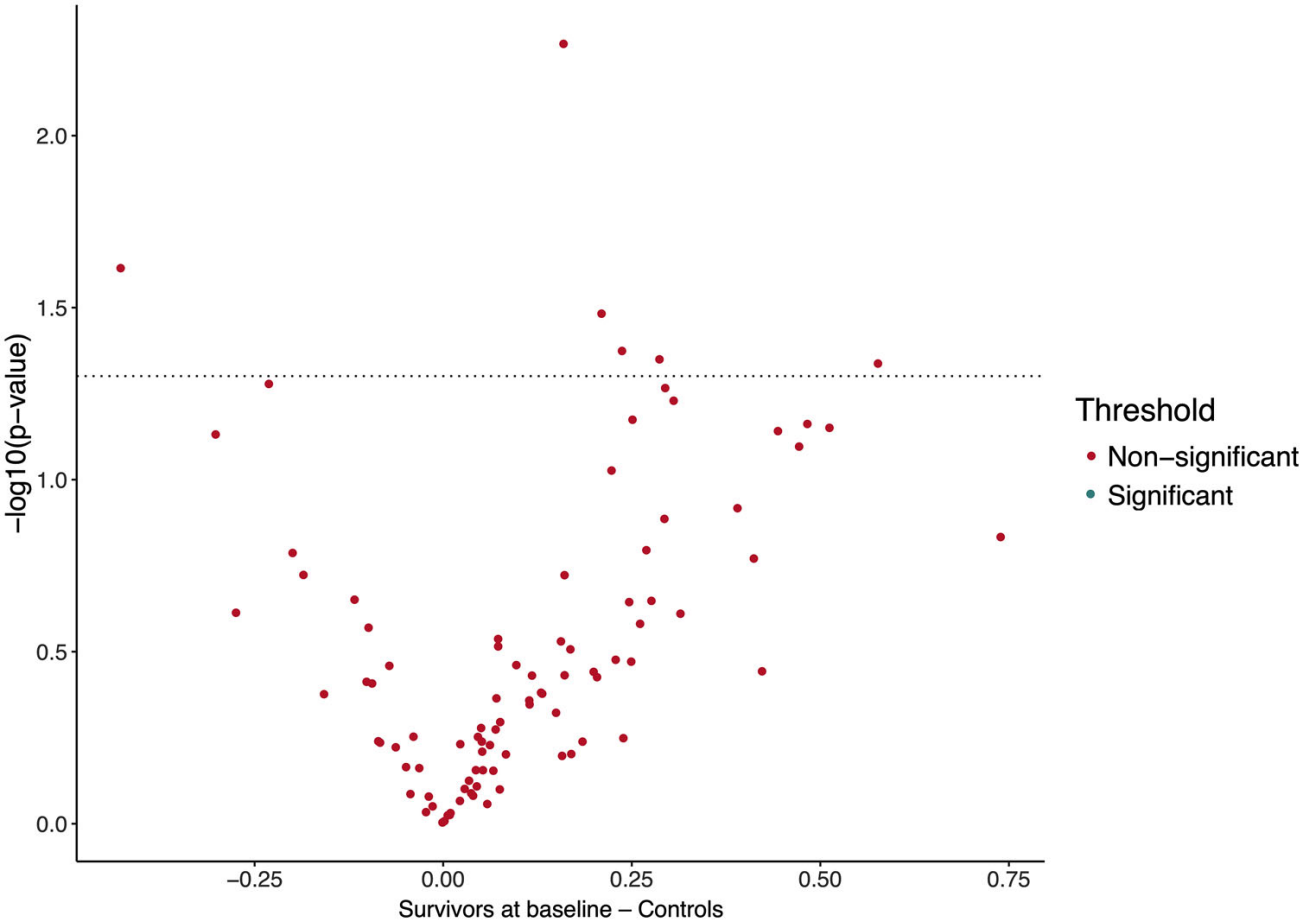
Volcano plot of the paired *t*‐test between the acute lymphoblastic leukemia (ALL) survivors at baseline (*N* = 21) and the controls (*N* = 21). No statistically significant differences in protein expression levels were found after correction with Benjamini–Hochberg method, which is represented by all the proteins being presented as red dots, that is, the corrected *p*‐values did not reach <0.05. The dotted line represents the uncorrected significance threshold of 0.05. On the *y*‐axis are log_10_ of *p*‐values and on the *x*‐axis is the log_2_ fold change between the two groups where a positive fold change indicates a lower protein level in the survivors than in the controls.

No statistically significant differences in the inflammatory protein concentrations at baseline between the long‐term survivors of ALL and their age‐ and sex‐matched controls were observed (Figure 3). No statistically significant differences in the smoking or alcohol consumption habits between the survivors at baseline and controls were identified (*p* = 0.50 and 0.90, respectively).

**FIGURE 4:**
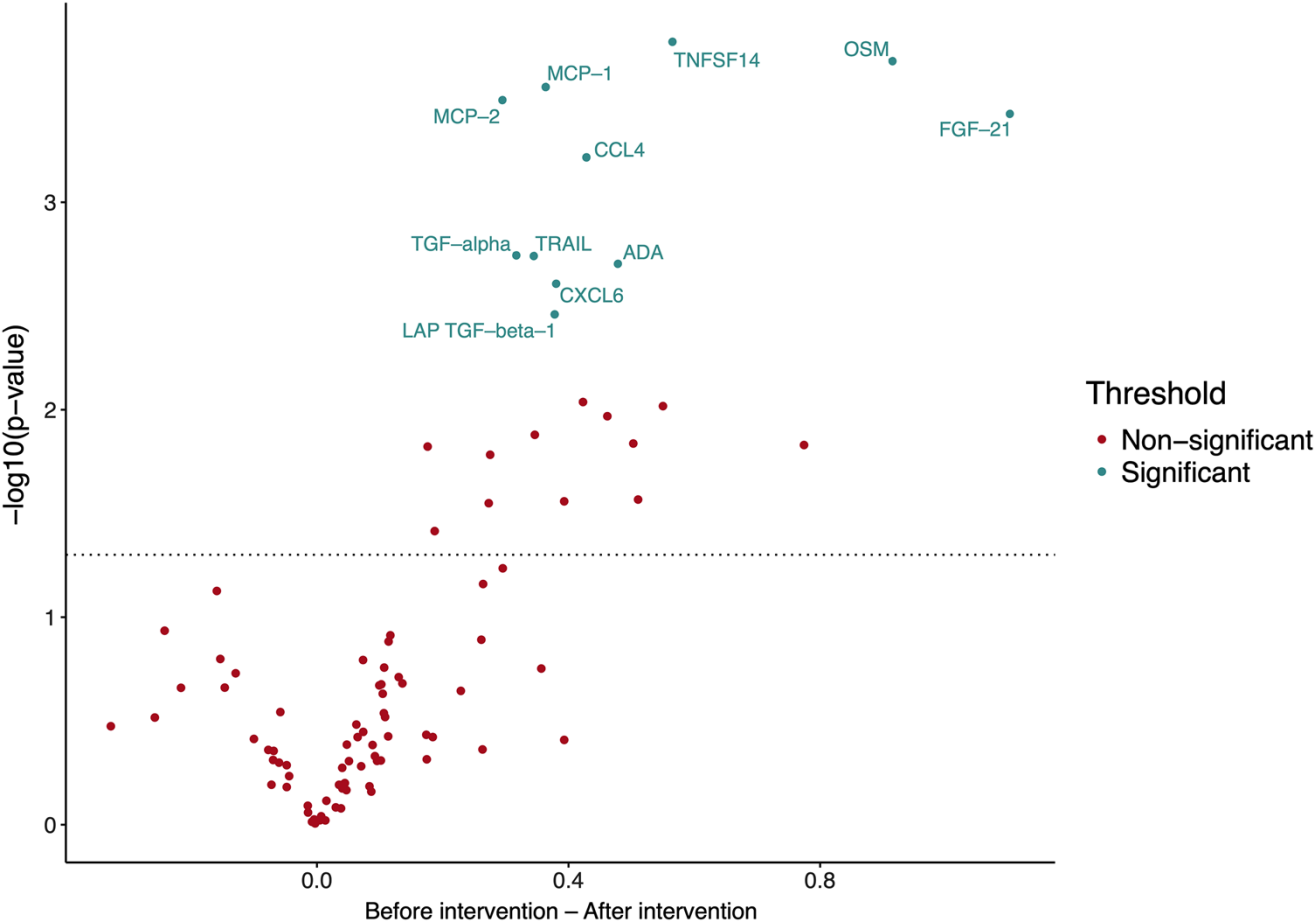
Volcano plot of the paired *t*‐test between the acute lymphoblastic leukemia (ALL) survivors at baseline (*N* = 17) and after intervention (*N* = 17). The proteins with statistically significant changes (after Benjamini–Hochberg method correction) in expression level between the two time points are labeled with names. The dotted line represents the uncorrected significance threshold of 0.05. On the *y*‐axis are log_10_ of *p*‐values and on the *x*‐axis is the log_2_ fold change between the two groups where a positive fold change indicates a decrease in the protein level during the exercise intervention.

**FIGURE 5:**
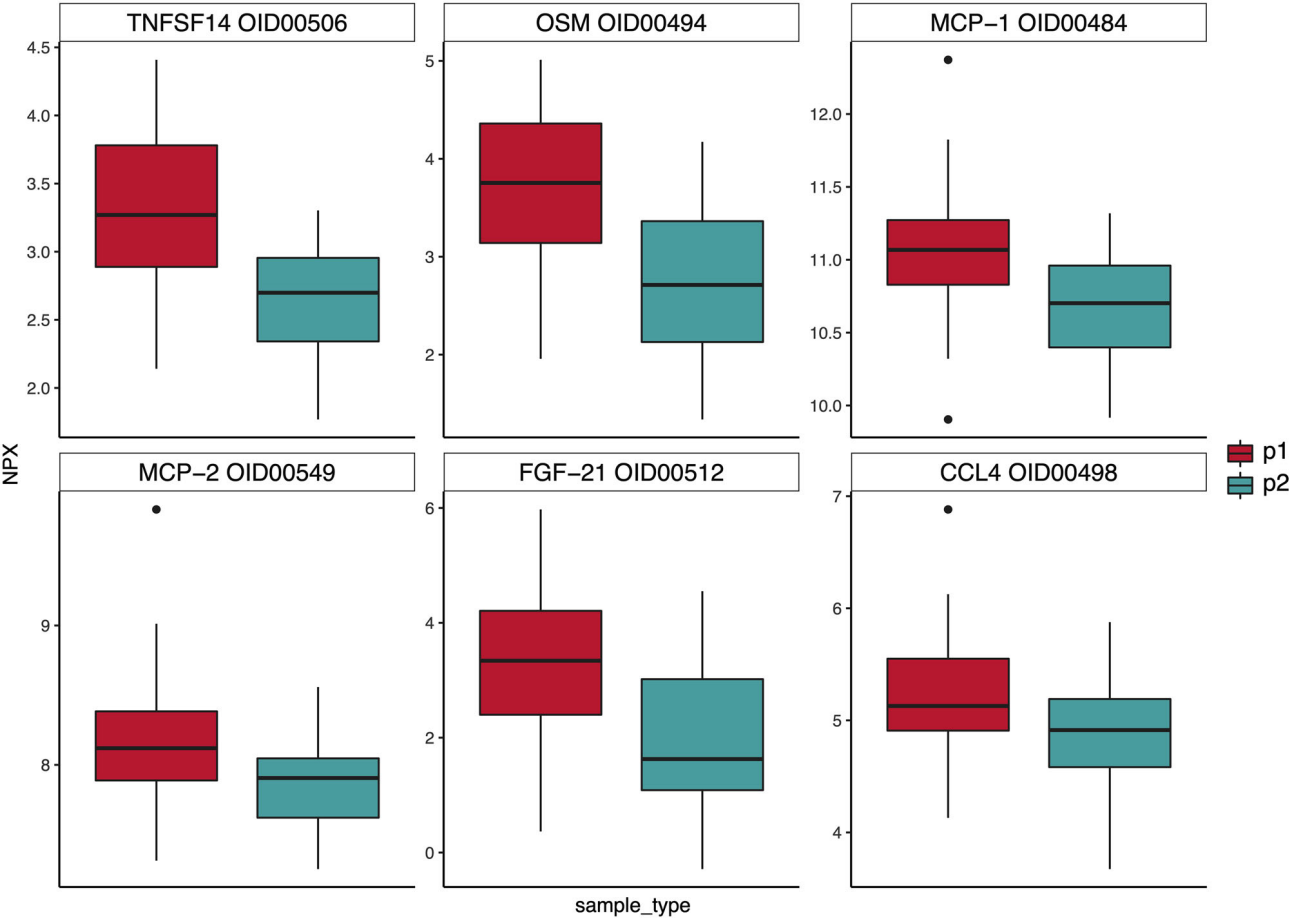
Boxplots of the six proteins with the most significant changes in protein expression levels following paired *t*‐tests between p1 (acute lymphoblastic leukemia [ALL] survivors at baseline, *N* = 17) and p2 (ALL survivors after intervention, *N* = 17). Normalized Protein eXpression (NPX) is the unit on all of the *y*‐axes and colors denote sample type (p1 or p2) on the *x*‐axes. NPX is in log_2_ scale and it is used only for relative quantification and the values, as such, can only be compared for the same protein across different samples analyzed in one project. The levels of all six proteins decreased during the exercise intervention. Protein names and their Olink identification numbers are displayed above the boxplots.

**FIGURE 6:**
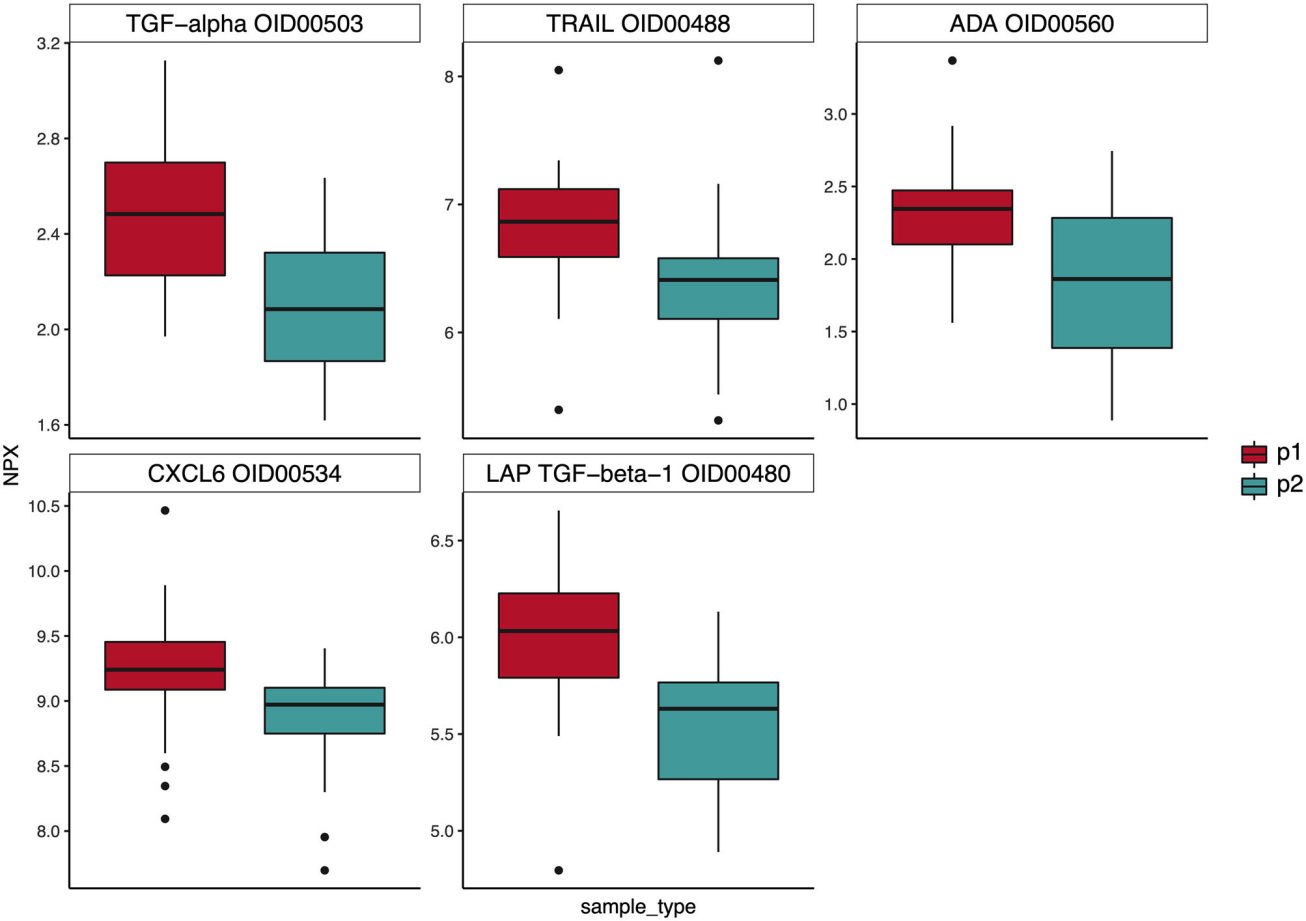
Boxplots of the other five proteins with statistically significant changes in protein expression levels following paired *t*‐tests between p1 (acute lymphoblastic leukemia [ALL] survivors at baseline, *N* = 17) and p2 (ALL survivors after intervention, *N* = 17). Normalized Protein eXpression (NPX) is the unit on all of the *y*‐axes and colors denote sample type (p1 or p2) on the *x*‐axes. NPX is in log_2_ scale and it is used only for relative quantification and the values, as such, can only be compared for the same protein across different samples analyzed in one project. The levels of all five proteins decreased during the exercise intervention. Protein names and their Olink identification numbers are displayed above the boxplots.

However, a statistically significant change in the inflammatory protein profile of the 17 ALL survivors who completed the physical exercise intervention was observed (Figure 4). Plasma concentrations of 11 out of 92 analyzed proteins were significantly lower post‐intervention than at baseline among ALL survivors (Figures 5 and 6). In descending order of uncorrected statistical significance, the 11 proteins included: tumor necrosis factor superfamily member 14 (TNFSF14), oncostatin M (OSM), monocyte chemoattractant protein 1 (MCP‐1), MCP‐2, fibroblast growth factor 21 (FGF‐21), chemokine (C‐C motif) ligand 4 (CCL4), transforming growth factor alpha (TGF‐α), tumor necrosis factor‐related apoptosis‐inducing ligand 10 (TRAIL), adenosine deaminase (ADA), chemokine (C‐X‐C motif) ligand 6 (CXCL6), and latency‐associated peptide transforming growth factor beta 1 (LAP TGF‐β1) (Table 1). The sizes of the protein levels’ mean log_2_ fold change are presented in Table 1 as well and they are seen on *y*‐axes of the boxplots in Figures 5 and 6. Descriptions of the functions of the 11 proteins are presented in Table S2. Our interpretation of the mechanisms of function of these 11 proteins is that TNFSF14, MCP‐1, CCL4, TRAIL, ADA, and LAP TGF‐β1 are involved in vascular inflammation. OSM, MCP‐2, TGF‐α, and CXCL6 are likely to have an association with endothelial dysfunction and some are mostly or additionally involved in IR (MCP‐1, FGF‐21, TRAIL, and ADA) or insulin metabolism (TNFSF14/LIGHT).

**TABLE 1:** Corrected and uncorrected *p*‐values of the 11 proteins with significantly changed expression levels after the intervention

Protein	Corrected *p*‐value	Uncorrected *p*‐value	Log_2_ fold change (95% CI)
TNFSF14/LIGHT	0.0069	0.000169	0.57 (0.32–0.81)
OSM	0.0069	0.000209	0.92 (0.51–1.32)
MCP‐1/CCL2	0.0069	0.000278	0.36 (0.20–0.53)
MCP‐2/CCL8	0.0069	0.000322	0.30 (0.16–0.43)
FGF‐21	0.0069	0.000375	1.10 (0.58–1.62)
CCL4/MIP‐1‐β	0.0093	0.000608	0.43 (0.21–0.64)
TGF‐α	0.0202	0.0018	0.32 (0.14–0.50)
TRAIL/TNFSF10	0.0202	0.00182	0.34 (0.15–0.54)
ADA	0.0202	0.00198	0.48 (0.20–0.75)
CXCL6	0.0227	0.00247	0.38 (0.16–0.61)
LAP TGF‐β1	0.0290	0.00347	0.38 (0.14–0.61)

*Note*: *p*‐Values <0.05 after correction with the Benjamini–Hochberg method were considered statistically significant. The sizes of the protein levels’ mean change in log_2_ scale with 95% confidence intervals (CI). A difference of 1 equates to halving of the protein level.

Abbreviations: ADA, adenosine deaminase; CCL, chemokine (C‐C motif) ligand; CXCL, chemokine (C‐X‐C motif) ligand; FGF‐21, fibroblast growth factor 21; LAP TGF‐β1, latency‐associated peptide transforming growth factor beta 1; MIP‐1‐β, macrophage inflammatory protein‐1‐beta; MCP, monocyte chemoattractant protein; OSM, oncostatin M; TGF‐α, transforming growth factor alpha; TNFSF14/LIGHT, tumor necrosis factor superfamily member 14; TRAIL/TNFSF10, tumor necrosis factor‐related apoptosis‐inducing ligand 10.

## 4 DISCUSSION

In the present study, we found a decrease in 11 proteins related to either vascular inflammation, IR, or both after the exercise intervention in long‐term survivors of ALL. In the original study [17, 18], endothelial dysfunction and IR were also significantly ameliorated after the intervention.

At baseline, the long‐term ALL survivors did not differ in their plasma inflammatory protein levels compared to their age‐ and sex‐matched controls. This was unexpected in light of reports of ALL survivors being at risk for inflammation [38]^–^[40] and accelerated aging [24] and considering the diseases affiliated with these conditions, such as MetS [41]. Using an inflammatory protein panel this wide, not many similar results have been reported earlier. Interpretation of the results is complicated by the fact that the survivors had worse fitness (VO_2peak_) than the controls, but had similar levels of prior physical activity compared to the controls in the original study [12]. In addition, the original study [12, 17] found no significant differences in, for example, BMI, systolic blood pressure, fasting glucose, high‐density lipoprotein cholesterol, or triglycerides between the survivors and controls at baseline (Table S1) and we found similar alcohol drinking habits in the two groups and no statistically significant differences in the smoking habits were found either. Anthracycline chemotherapy has been shown to have a dose‐dependent correlation with VO_2peak_ among survivors of ALL [8], which may be the explanation for poor exercise capacity among ALL survivors, despite no inflammatory differences between the survivors and controls. In the original study [12], the ALL survivor cohort was found to be similar to unparticipating survivors in leukemia‐related factors (treatment intensity and protocol) and regarding, for example, self‐reported BMI and level of physical activity, which undermines the possibility of attributing this result to an unrepresentative sample. The survivors of childhood ALL were adolescents and young adults (ages 16–30 years) at the time of the study, so the lack of differences in protein expression levels at baseline compared to healthy controls may be due to the long duration of time it takes to develop a pathological disease state, such as MetS or CVD. Rather than inflammatory factors, the original study's observed worse VO_2peak_ in the survivors may also be explained by impairments in pulmonary, autonomic and muscle function, as suggested by recent studies [11]. Furthermore, the control group included seven of 21 overweight (BMI > 25) subjects and the patient group included eight of 21 subjects (Table S1), which likely narrows their proteomic differences. Ten of the 21 controls had a below‐average physical condition compared to 16 of 21 in the patient group, when physical condition was measured as peak oxygen uptake (VO_2peak_) in proportion to weight and classified by age and gender reference values [17]. Additionally, a large inter‐individual variation in protein profiles compared to rather stable intra‐individual levels [42] may have led to a lack of power in the analysis between the survivors and controls.

Of the 11 proteins that improved after the exercise intervention, TNFSF14/LIGHT decreased most significantly during the exercise intervention compared to baseline. This protein has been found to be involved in endothelial inflammation [43] and impaired insulin secretion [44]. The ALL survivors had a significant improvement in their endothelial function as measured by the left common carotid artery intima media thickness and flow‐mediated dilation of the left brachial artery in the original study [18]. Our findings support the perception of LIGHT being involved in endothelial inflammation and atherogenesis, and that increased exercise may decrease these vascular pathologies. The survivors’ insulin metabolism, that is IR, improved during the intervention and the decrease in LIGHT expression levels is in line with the improvement seen in insulin metabolism.

The function of the protein OSM is not well defined. Regarding its pro‐inflammatory versus anti‐inflammatory effects, more documentation of the pro‐inflammatory effects exists in humans, at least when it comes to vascular injury [45]. Endothelial cells express high levels of OSM receptors, making them one of the primary target cells for OSM, which is suggested to have an indirect ability to increase vascular permeability and perivascular infiltration of immune cells at the sites of tissue damage [45]. The decrease in OSM during the intervention further supports its role as pro‐inflammatory in endothelial inflammation.

MCP‐1 plays a role in endothelial dysfunction caused by inflammation [46], and significantly elevated levels have been reported in obese insulin‐resistant adults [47]. The decrease in MCP‐1 is in agreement with the clinical findings of the original study [17] in both these regards. MCP‐2/CCL8 is known to inhibit the chemotactic activity of MCP‐1 [46], whose levels decreased during the intervention. The mechanisms by which MCP‐2 levels decreased during the intervention are not as clear, but they are likely to be related to attenuated inflammation and linked to the decrease seen in MCP‐1 [48]. Though, even MCP‐2 has recently been shown to be present in the endothelium of advanced human carotid plaques [49] suggesting its involvement in endothelial dysfunction, too.

We found support for the survivors’ improved insulin metabolism even through FGF‐21, whose plasma expression levels decreased during the intervention. This cytokine is related to IR and MetS even in pediatric populations [47] with elevated levels suggesting resistance to it. Even involvement in β‐cell failure has been suggested [47]. Due to the cytokine's stimulatory role in glucose uptake, there have been promising findings in the form of dyslipidemia improving outcomes from several of the clinical trials that are developing long‐acting FGF‐21 analogs [50]. Altogether, the literature indicates that FGF‐21 in humans is an insulin‐dependent hormone with primarily postprandial release in addition to exercise‐induced release from mainly the liver [50]. In keeping with our MCP‐1 data and the original study's significantly reduced IR, the exercise program led to reduced levels of FGF‐21, which is in alignment with a reported positive correlation between plasma insulin and FGF‐21 levels [51].

The recurring association between a marker of vascular inflammation (Table S2) and the improvement in our survivors’ endothelial function was seen in the case of CCL4, too, as its levels decreased during the intervention. A study on children with untreated primary hypertension also discovered CCL4/macrophage inflammatory protein‐1‐beta to be significantly elevated when compared to healthy peers [52]. Furthermore, patients with MetS have also been reported to have significantly elevated levels of CCL4 and its receptor C‐C chemokine receptor 5 (CCR5) [53]. The same study reported both CCL4 and CCR5 levels to significantly reduce in response to a low‐dose statin treatment, of which the former's reduction has been reported by others as well [54], albeit in a cohort of patients with coronary artery disease.

It might be that TGF‐α has no direct association with endothelial dysfunction or CVD (Table S2), but the significantly lowered levels of the protein in our study during the intervention could be explained indirectly via its effects on other inflammatory proteins [55, 56].

Our survivors had also lower plasma expression levels of TRAIL after the intervention than at baseline. TRAIL has previously been suggested to have a protective role in endothelial dysfunction [57] and in ischemic vascular diseases based on clinical evidence [58], but opposite effects of TRAIL have also been reported [59, 60]. Forde et al. [61] reviewed both the protective and pro‐atherogenic results on TRAIL and concluded that TRAIL has pleiotropic effects on the vasculature. We report a decrease in TRAIL levels in childhood ALL survivors after the exercise intervention, suggesting that in young subjects TRAIL levels decrease when endothelial dysfunction is alleviated. Based on previous literature and our results, our hypothesis is that TRAIL has different roles at different stages of atherosclerosis.

We saw as well a significant reduction in the levels of ADA from baseline to post‐intervention, which is in line with the significant evidence of adenosine being involved in vascular barrier dysfunctions and endothelial dysfunction (Table S2). How to affect adenosine pharmacologically in the context of CVD has been previously discussed [62] and clopidogrel is already an established example of a drug affecting the purinergic metabolism. Instead, we exhibit evidence of our previous 16‐week exercise program affecting this metabolism in the form of lowered levels of ADA without any medication. Additionally, receptor inhibitors of ADA, dipeptidyl peptidase‐4 inhibitors, are established anti‐diabetics.

No studies on endothelial dysfunction and CXCL6 seem available in the literature (Table S2), but our results showing significantly reduced levels of CXCL6 during the intervention suggest it may have a role as a biomarker of endothelial inflammation, as the endothelial function had improved at the post‐intervention phase. A very recent study [63] shows CXCL6 to be a new specific marker for cardiosphere‐derived cells which are cells with characteristics of inflammatory cells. They are found in human cardiac biopsies and have disease‐modifying capabilities in, for example, cardiac conditions.

TGF‐β has been suggested to regulate atherogenesis even in humans, and in animals it is reported to have anti‐atherosclerotic properties, mainly through inhibition of T cells with atherosclerotic functions, but possibly even by directly regulating endothelial cells and macrophages among other cell types [64]. We found the plasma LAP TGF‐β1 expression levels to be significantly lower after the exercise program than before it, which indicates an increase in the active form (TGF‐β1) as the latent form (Table S2) reduced. Thus, we suggest TGF‐β to be active in resolving endothelial inflammation. The active form was not measured in this study.

In summary, the long‐term survivors of ALL did not have any differences in their inflammatory burden compared to their peers in our cohort of 21 former patients and 21 healthy controls. Instead, a home‐based 16‐week exercise intervention resulted in reduced expression profiles of low‐grade inflammation biomarkers in the plasma of 17 ALL survivors who had completed the program. The 11 proteins that were significantly decreased in this study are involved most often in vascular inflammation, like in the case of TNFSF14, MCP‐1, CCL4, TRAIL, ADA, and LAP TGF‐β1. Some of the identified proteins do not have a clear role in endothelial inflammation, but we consider OSM, MCP‐2, TGF‐α, and CXCL6 to have an association with endothelial dysfunction when the proteomic results are considered together with our clinical data (Table S1). Some of the proteins were found to be mostly or additionally related to IR, like in the case of MCP‐1, FGF‐21, TRAIL, and ADA, or insulin metabolism in the case of TNFSF14/LIGHT. Although the roles of some of the identified proteins were more difficult to interpret based on available data, the observed changes in their plasma expression levels after the intervention reflect a healthier inflammatory profile.

Despite the fact that no change was observed in some proteins related to endothelial inflammation (such as fractalkine/chemokine (C‐X3‐C motif) ligand 1, colony stimulating factor‐1, or hepatocyte growth factor) following completion of the intervention, we can state that a home‐based exercise program does alleviate cardiovascular pathology on a biomarker level in a population with similar baseline inflammatory profiles as its healthy peers. We consider there to be room to increase the number of not only randomized controlled trials, but even these types of exploratory studies among ALL survivors as the population is at risk for premature morbidity, exercise is a potential modifier of this risk, and the population can benefit from potential early detection of localized pathologies which precede systemic effects.

Limitations of this study were the relatively small sample size and drop‐out of four subjects before completion of the intervention. The lack of controlled diet was also a limitation as high‐fat diets have been shown to influence systemic low‐grade inflammation [65] and not having controlled the exact alcohol consumption amounts might have also contributed to the lack of differences in survivors versus controls. If we had had the possibility to compare changes in the controls after an exercise intervention, it could have helped verify some of the inflammatory changes that we saw in the survivors, but also provide further base for making conclusions about the survivors’ initial health, as it now seems to have been similar to the healthy controls’ at baseline, at least regarding their inflammatory profiles. Furthermore, five subjects had received cranial irradiation, leading to a heterogeneity of the survivor group.

Future studies should involve larger sample sizes for greater statistical power and additional interventions to reduce the burden of low‐grade inflammation on these young adults. Studies with more recently treated patients should also be performed to better evaluate the potential consequences of today's more intensive chemotherapy‐only treatment on ALL survivors’ inflammatory profiles compared to their healthy peers. The improvements seen in inflammatory protein profiles after our previous exercise intervention further emphasize the role of exercise in mitigating the inflammatory state and risk of CVD among survivors of ALL. We would like to suggest that these kinds of positive effects following exercise could be achieved by the general public as well, as the survivors’ inflammatory profiles did not differ from their controls’ profiles at baseline.

## CONFLICTS OF INTEREST

The authors declare they have no conflicts of interest. A preprint of this article's previous version is published with DOI 10.21203/rs.3.rs‐961004/v1 on Research Square.

## ETHICS STATEMENT

This add‐on study falls under the original study's ethical permission from the Commission on Ethics of Southwest Finland Hospital District (reference number 45/2007) and was performed in accordance with the Declaration of Helsinki.

## PATIENT CONSENT STATEMENT

Written informed consent from each participant was obtained as part of the original study.

## Supporting information

SUPPLEMENTAL TABLE S1 Compiled data from the earlier studies that was now utilized. A representation of the main outcomes from the original studies. AUC, area under the curve; BMI, body mass index; F, fasting; FMD, flow‐mediated dilation; HOMA‐IR, homeostatic model assessment for insulin resistance; MET, metabolic equivalent of task where 12 minutes of moderate intensity activity corresponds to 1 MET hour/week; *N*, number; SD, standard deviation; VO_2_, oxygen consumption. ^a^The variable was categorized and the difference between the groups was analyzed with cross‐tabulation and Fischer's exact test.

SUPPLEMENTAL TABLE S2 Descriptions of the functions of the 11 proteins with significantly lowered expression levels, presented in a descending order of uncorrected statistical significance.

SUPPLEMENTAL FIGURE S1 Distribution boxplot of all 59 samples colored by groups: p1 (acute lymphoblastic leukemia [ALL] survivors at baseline), p2 (ALL survivors after intervention), and v1 (controls) and expressed in the Normalized Protein eXpression (NPX) unit. No outliers were identified.

SUPPLEMENTAL FIGURE S2 Principal component analysis (PCA) plot of all 59 samples colored by sample type p1 (acute lymphoblastic leukemia [ALL] survivors at baseline), p2 (ALL survivors after intervention), and v1 (controls). No clear outlier samples were identified.

## Data Availability

The data that support the findings of this study are available from the corresponding author upon reasonable request.
