# Supplementary figures and images for: Inflammatory biomarkers after an exercise intervention in childhood acute lymphoblastic leukemia survivors
Source: EJHaem. 2022 Sep 29;3(4):1188–200. doi: 10.1002/jha2.588 (PMC9713025; doi:10.1002/jha2.588)

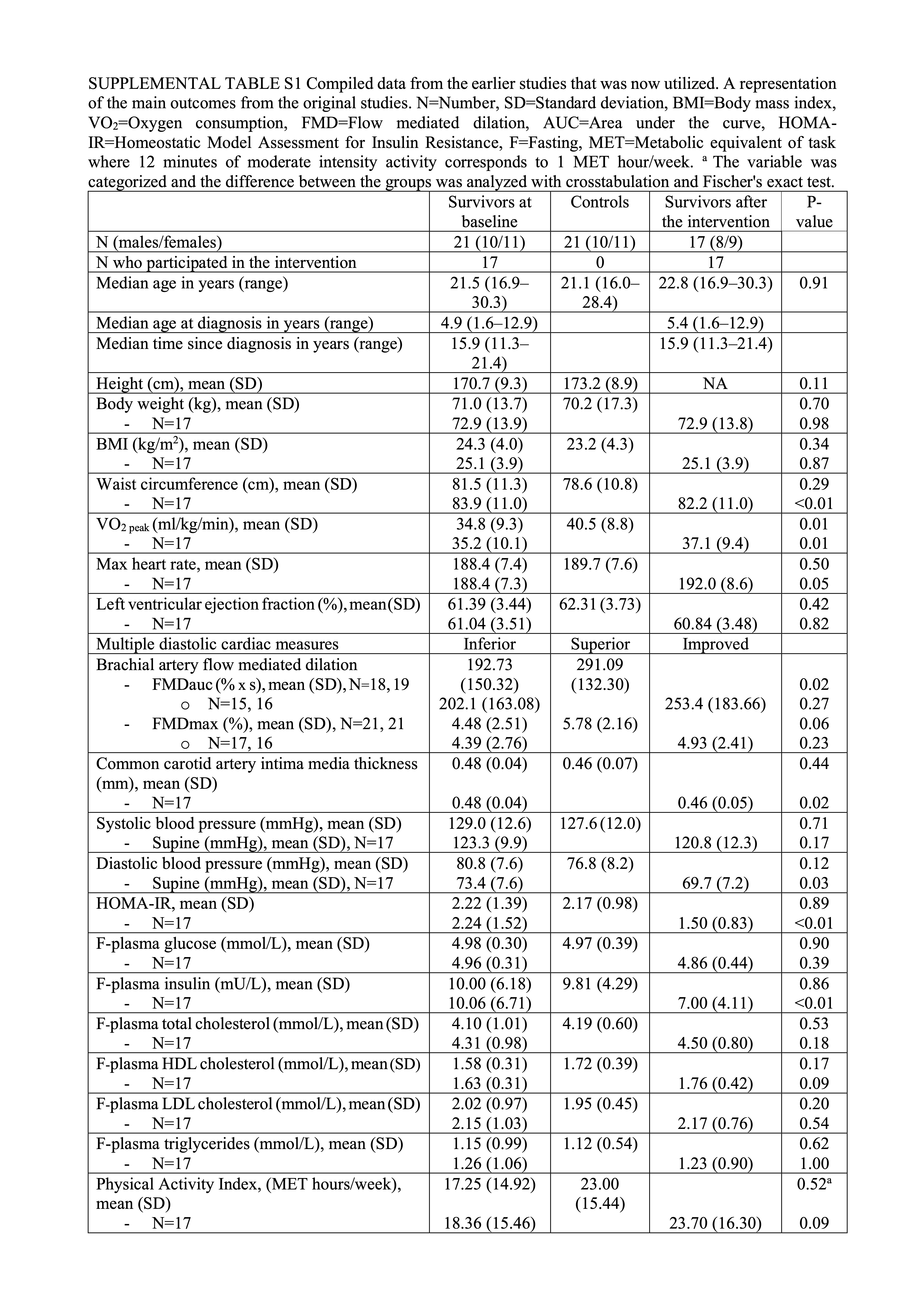

Supplement: Supplementary file 1 — SUPPLEMENTAL TABLE S1 Compiled data from the earlier studies that was now utilized. A representation of the main outcomes from the original studies. AUC, area under the curve; BMI, body mass index; F, fasting; FMD, flow‐mediated dilation; HOMA‐IR, homeostatic model assessment for insulin resistance; MET, metabolic equivalent of task where 12 minutes of moderate intensity activity corresponds to 1 MET hour/week; N, number; SD, standard deviation; VO2, oxygen consumption. aThe variable was categorized and the difference between the groups was analyzed with cross‐tabulation and Fischer's exact test. [file JHA2-3-1188-s003.jpg]

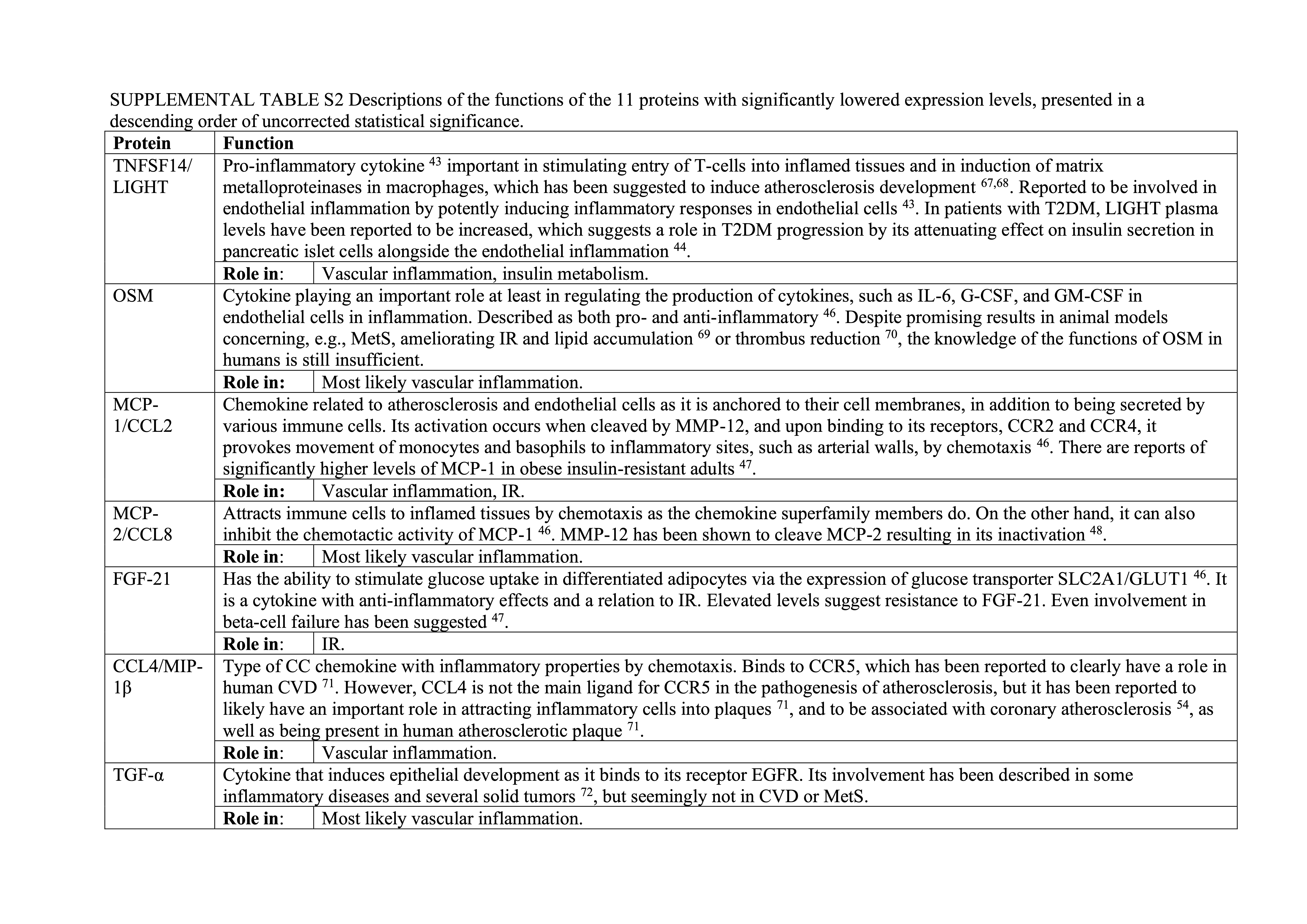

Supplement: Supplementary file 2 — SUPPLEMENTAL TABLE S2 Descriptions of the functions of the 11 proteins with significantly lowered expression levels, presented in a descending order of uncorrected statistical significance. [file JHA2-3-1188-s004.jpg]

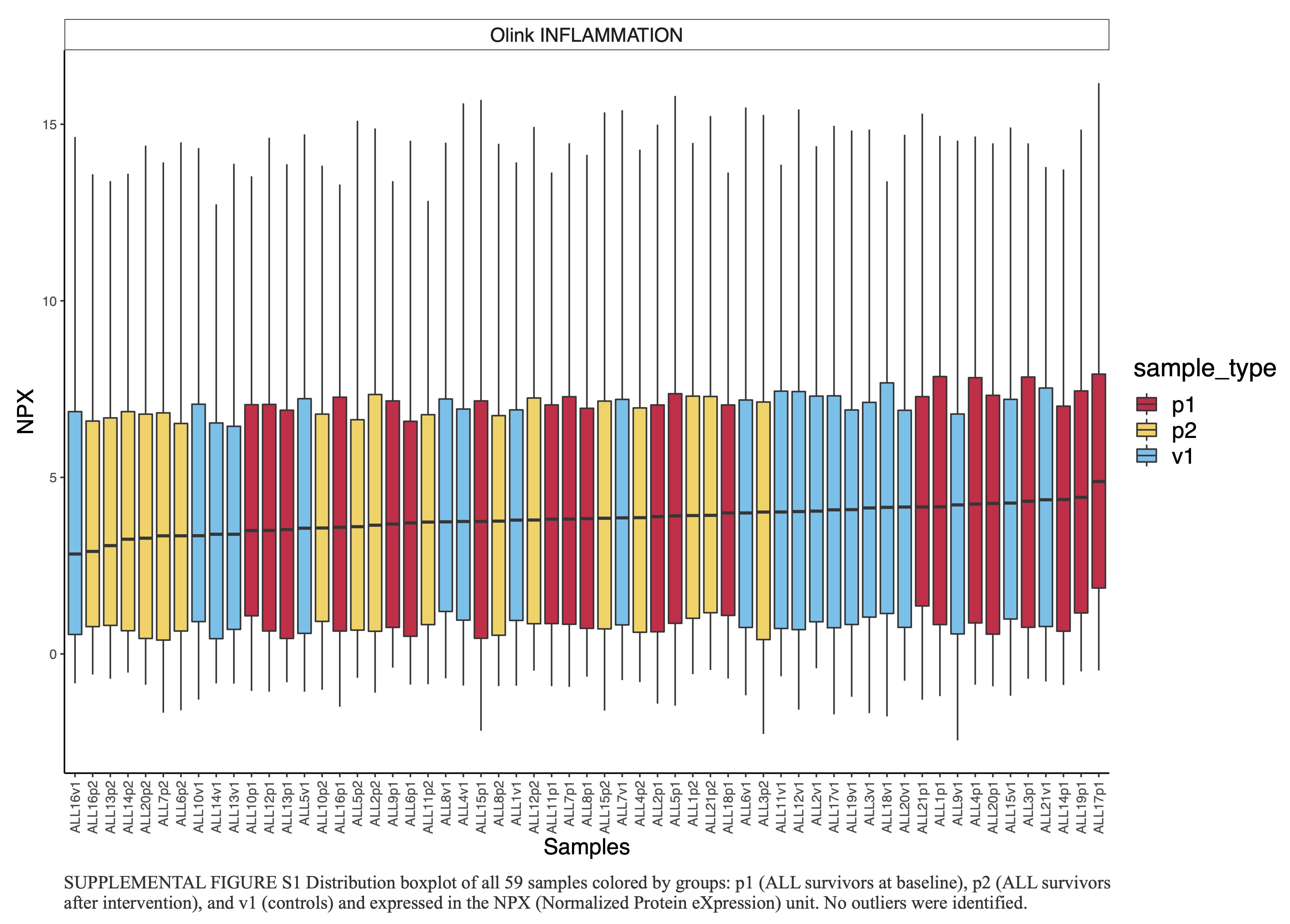

Supplement: Supplementary file 3 — SUPPLEMENTAL FIGURE S1 Distribution boxplot of all 59 samples colored by groups: p1 (acute lymphoblastic leukemia [ALL] survivors at baseline), p2 (ALL survivors after intervention), and v1 (controls) and expressed in the Normalized Protein eXpression (NPX) unit. No outliers were identified. [file JHA2-3-1188-s002.jpg]

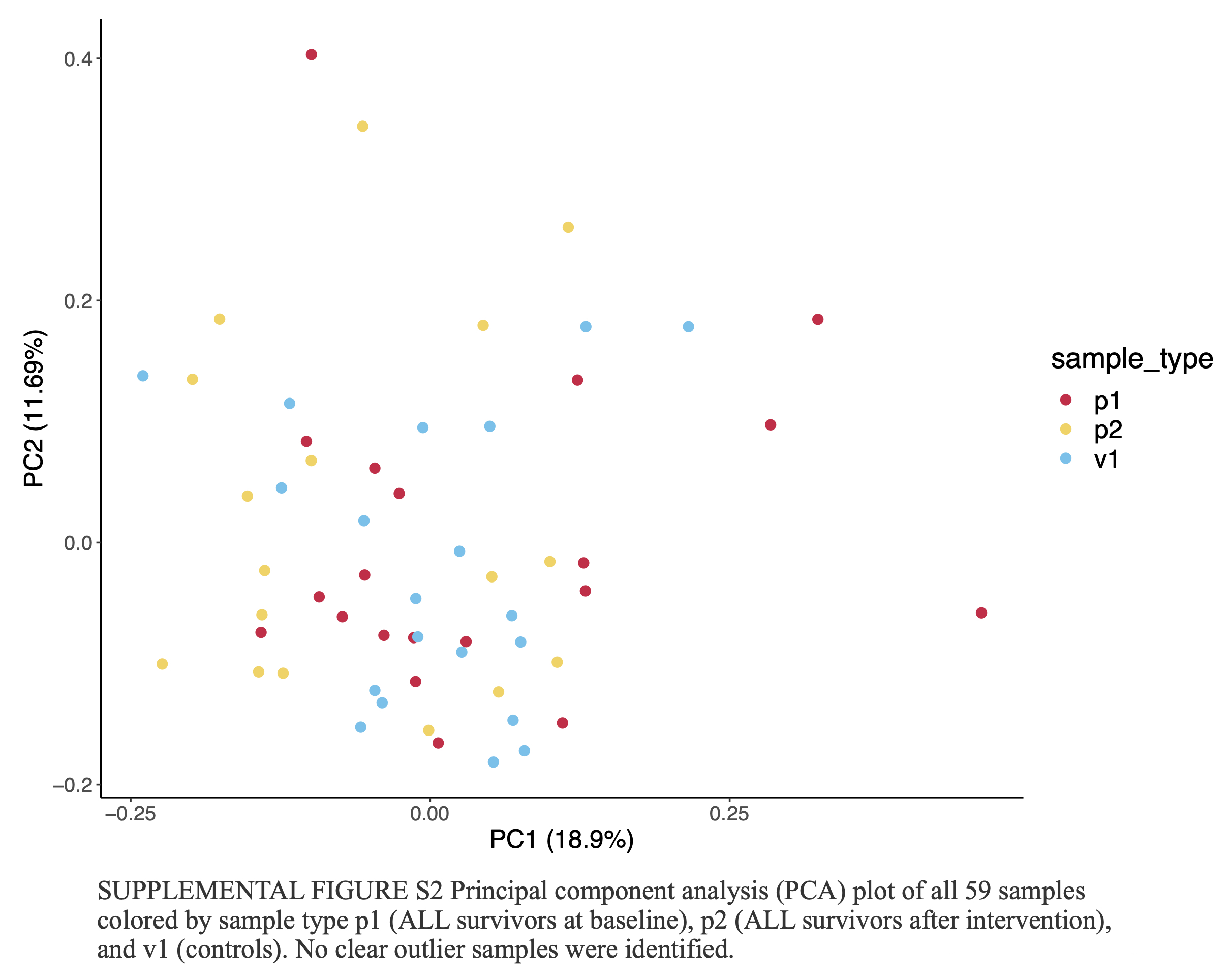

Supplement: Supplementary file 4 — SUPPLEMENTAL FIGURE S2 Principal component analysis (PCA) plot of all 59 samples colored by sample type p1 (acute lymphoblastic leukemia [ALL] survivors at baseline), p2 (ALL survivors after intervention), and v1 (controls). No clear outlier samples were identified. [file JHA2-3-1188-s001.jpg]
